# Massachusetts

**Published:** 1899-06

**Authors:** H. S. Lewis

**Affiliations:** Secretary


Secretary.

### MASSACHUSETTS VETERINARY ASSOCIATION.

The fifteenth annual meeting was held April 26th, at the Parker House, Boston. The following members were present: Drs. Beckett, Burr, Clark, Cronan, Emerson, Etienne, Frothingham, Harrington, Howard, La Baw, Lee, Lewis, McLaughlin, Osgood, Parker, Penniman, Peters, Peterson, Pierce, Plaskett, Rodgers, Williams, Winslow, Winchester, and Stickney. W. O. Underwood, A.B., Lecturer on Warranty and Evidence at the Veterinary Department of Harvard University; Leander F. Herrick, of the Massachusetts Cattle Commission; James Kimball, of the Board of Health, Springfield, Mass.

At the business meeting the following officers were elected for the ensing year: President, Langdon Frothingham, M.D.V.; First Vice-President, Howard P. Rodgers, M.D.V.; Seocnd Vice-President, Daniel Emerson, M.D.V.; Treasurer and Secretary, Henry S. Lewis, M.D.V.; Executive Committee—W. L. La Baw, D.V.S.; George Lee, D.V.S.; E. T. Harrington, M.D.V.; G. P. Penniman, D.V.S., and B. D. Pierce, M.R.C.V.S.

At the close of the business meeting the Association adjourned to the banquet-hall, where the Toast-master, Dr. J. F. Winchester, called on the following gentlemen: W. O. Underwood, J. M. Parker, L. F. Herrick, Langdon Frothingham, James Kimball, J. R. McLaughlin, Austin Peters, P. J. Cronan, L. H. Howard, Alexander Burr, B. D. Pierce, H. S. Lewis, and J. H. Stickney.

H. S. LEWIS,
Secretary.

